# From Gut to Shot: Microbiome-Guided Strategies to Improve Vaccine Responses in Food Animals

**DOI:** 10.3390/vaccines14040327

**Published:** 2026-04-07

**Authors:** Muhammad Saeed Akhtar, Wajid Zaman

**Affiliations:** 1 Department of Chemistry, Yeungnam University, Gyeongsan 38541, Republic of Korea; msakhtar@yu.ac.kr; 2 Department of Life Sciences, Yeungnam University, Gyeongsan 38541, Republic of Korea

**Keywords:** vaccine immunogenicity, gut microbiome, immunometabolism, short-chain fatty acids, nutritional immunology, biomarkers

## Abstract

Vaccine performance in livestock and poultry often varies under field conditions. Conventional explanations, such as handling errors, cold-chain failures, or antigen mismatch do not fully account for inconsistent immunogenicity and durability. Increasing evidence suggests that the gut microbiome acts as an upstream regulator of vaccine responses through microbial structural signals and metabolites that shape antigen presentation, B-cell metabolism, and inflammatory tone. Early life microbiome disruption can impair antibody responses to multiple vaccines, highlighting a plausible causal role for dysbiosis in suboptimal vaccine efficacy. Microbiota-derived metabolites, particularly short-chain fatty acids (SCFAs), can influence B-cell differentiation and antibody production through metabolic and epigenetic pathways. However, these effects are dose- and context-dependent, highlighting the need for controlled translation rather than generalized assumptions that higher SCFA levels are beneficial. This review synthesizes microbiome–immunometabolism pathways relevant to vaccine responses in food animals and assesses practical nutritional and microbiome-targeted strategies, such as amino acids, trace minerals, organic acids, phytogenics, and postbiotics, that may modulate these pathways to improve outcomes. We also propose field-deployable biomarker panels that combine immune readouts with inflammation- and microbiome-linked metabolite proxies to stratify likely responders, monitor intervention effects, and improve trial comparability. Finally, we outline translational study designs that connect microbiome shifts to protective immune endpoints and performance outcomes, enabling evidence-based integration of microbiome-informed strategies into vaccination programs for poultry, with broader conceptual relevance to other food animals.

## 1. Introduction

Vaccine programs are central to disease control in modern livestock and poultry production. However, variability in immune responses remains a persistent field challenge, even when vaccines are appropriately stored, administered, and matched to circulating pathogens [1,2]. This variability is not trivial: inconsistent vaccine ‘take’, that is, the measurable induction of the expected post-vaccination immune response, and durability can result in uneven flock or herd protection, sporadic outbreak clusters, and increased therapeutic antimicrobial use driven by uncertainty and secondary infections [3,4]. A key limitation of conventional explanations is that they often treat vaccine response as a property of the antigen and the host in isolation. However, production systems impose strong and fluctuating pressures, such as diet transitions, stocking density, stress, coinfections, and routine antimicrobial exposures, that reshape immune readiness, defined here as the pre-vaccination physiological and immunological state that supports effective antigen presentation and adaptive priming [5,6,7]. Consequently, vaccine response is frequently an emergent property of the animal’s broader physiological state rather than as a fixed trait.

A growing body of research indicates that the gut microbiome is a major upstream driver of this physiological state, influencing both baseline immune tone and the capacity to mount vaccine-induced adaptive immunity [8,9]. Human and animal studies increasingly link microbiome composition and function to vaccine immunogenicity and durability, with microbial metabolites and bile acids repeatedly implicated as mediators that bridge microbial ecology and host immune programming [10,11]. Importantly, this does not imply that the microbiome solely determines vaccine responses. Rather, it is a plausible “hidden variable” that can explain why animals with similar genetics and management conditions can respond differently to identical vaccines.

Food animals provide a particularly relevant setting for microbiome-informed vaccination because early life in these species is characterized by rapid microbiome assembly and immune maturation, often occurring alongside management interventions that can disrupt microbial ecology (metaphylaxis, anticoccidials, abrupt feed changes) [9,12]. Experimental evidence from mammals shows that early life antibiotic-driven dysbiosis can impair antibody responses to multiple vaccines, highlighting a causal pathway through which microbiome disruption may translate into weaker vaccine outcomes [13]. In poultry, recent mechanistic research also supports the existence of a microbiome–immune–metabolism axis, including gut–lung crosstalk, which is highly relevant for respiratory vaccines and complex production environments [14].

Therefore, this review addresses a practical question: how can insights from microbiome science be translated into implementable strategies to improve the consistency of vaccine responses in food animals, with primary emphasis on poultry vaccination systems? We synthesize current information on mechanistic pathways, such as how microbial signals and metabolites shape immunometabolism, evaluate practical nutritional and microbiome-targeted levers with realistic constraints, propose field-deployable biomarker panels to monitor progress, and outline trial designs that link microbiome changes to protection and performance. The goal is not to replace established vaccination principles, but rather to add a biologically grounded layer that helps explain and reduce response variability under field conditions.

## 2. Mechanistic Basis: How the Microbiome Shapes Vaccine Responses

Microbiome effects on vaccine responsiveness arise through two broad mechanisms: microbial structural cues calibrate innate immune readiness and antigen presentation, and microbe-derived metabolites reprogram immune cell metabolism and differentiation. Innate priming is fundamental because vaccine immunogenicity depends on timely antigen sensing, dendritic cell maturation, and the quality of T-helper and germinal center responses that follow. When microbial cues are absent or distorted, such as after antibiotic-driven dysbiosis, adaptive responses may become blunted or dysregulated, with measurable reductions in antibody responses reported in early life disruption models [15]. This provides a plausible mechanistic link between perturbations in production systems and variability in vaccine performance.

**Figure 1:**
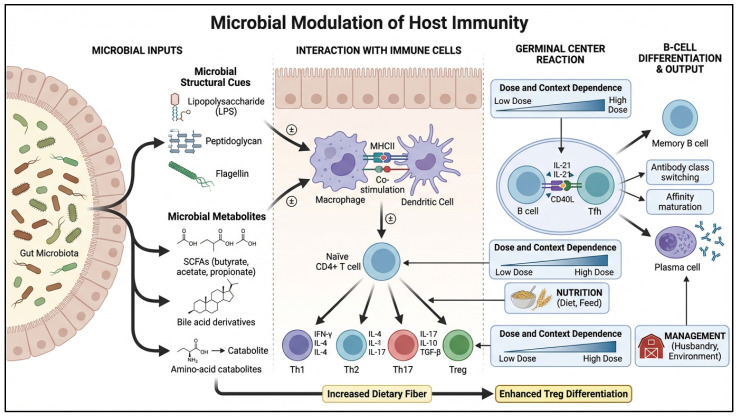
Microbiome–immunometabolism pathways influencing vaccine uptake. Conceptual schematic showing how microbial structural cues and metabolites shape innate and adaptive immune responses that influence vaccine uptake, including T-cell differentiation, germinal center activity, and B-cell outcomes. Dose-, diet-, and management-dependent effects are also highlighted. Created using https://www.figurelabs.ai, accessed on 3 March 2026.

Metabolites introduce a second layer by shaping immunometabolism, the metabolic programs that enable immune cells to proliferate, differentiate, and execute effector functions. Short-chain fatty acids (SCFAs), such as acetate, propionate, and butyrate, are among the most widely studied because they directly influence cellular energy pathways and can act as epigenetic modulators (Figure 1) [16]. Foundational studies shows that SCFAs can enhance B-cell metabolic activity and support antibody responses be increasing acetyl-CoA availability and broader metabolic remodeling [17]. These mechanistic insights are derived mainly from mammalian and in vitro immunology studies; direct validation in poultry vaccine systems remains limited. Additional studies show that SCFAs can influence B-cell differentiation and antibody production through histone deacetylase-related mechanisms, linking microbial fermentation products to humoral immunity at the level of gene regulation [18,19]. Again, these data provide biological plausibility but should not be interpreted as established avian or IBV-specific standards.

However, metabolite effects are not uniformly beneficial, and this nuance is critical for field translation; dose and context appear to be key determinants. Nikam et al. [20] report that sodium butyrate can suppress Tfh differentiation and impair germinal center responses under certain conditions, illustrating that SCFA supplementation may be beneficial or counterproductive depending on factors such as timing, concentration, and immune context. However, this dose- and context-dependent conclusion is based primarily on non-avian mechanistic studies, and direct confirmation in poultry IBV vaccination settings remains needed. Therefore, microbiome-guided vaccination should not be reduced to single-compound supplementation. Instead, it should be framed as the shaping of the metabolite environment to support appropriate innate priming and germinal center quality without excessive suppression or misdirection [21].

These mechanisms also interact with tissue axes relevant to food animals. Studies in poultry highlight the influence of the microbiome on systemic immune and metabolic signatures, including interactions along a gut–lung axis. This is directly relevant for respiratory vaccine responses and the reflects the practical reality that enteric and respiratory challenges co-occur in production systems [14]. Mechanistically, this supports an integrated view in which microbiome status affects not only local gut immunity but also systemic immune readiness that conditions vaccine uptake and durability.

## 3. Poultry Focus Case Study: Infectious Bronchitis Virus (IBV) Respiratory Vaccination Through a Microbiome–Immunometabolism Lens

In poultry production, IBV vaccination provides an ideal anchor system for microbiome-guided vaccination because vaccine uptake and durability can be inconsistent under field conditions, and protection failures are often multifactorial (variant circulation, coinfections, management pressure, immune immaturity) [22]. A controlled study using inbred chicken lines demonstrated that cecal microbiota composition is associated with the magnitude of the IBV vaccine response. The authors identified specific taxa (including *Escherichia*–*Shigella* operational taxonomic unit (OTUs)) that were negatively associated with stronger vaccine responses, along with immune correlates suggesting a role for TCRγδ^+^ T cells in mediating this interaction [23].

The mechanistic plausibility is strengthened by the chicken gut–lung axis, which provides a biologically coherent route linking gut microbiome composition, microbial metabolites, and respiratory vaccine responsiveness [14]. Saint-Martin et al. [24] highlights that gut microbiota modulation can strengthen mucosal immunity in chickens. It provides experimental evidence from germ-free versus conventional birds, indicating that the gut–lung axis is functional in chickens and may influence antiviral immunity and vaccine responsiveness. In this framework, fermentative products such as SCFAs generated in the gut can enter the circulation and contribute to systemic immunometabolic signaling, thereby shaping inflammatory tone and the functional state of immune cells beyond the intestine. In the respiratory tract, this systemic signaling may influence the readiness of antigen-presenting cells, interferon-linked innate responses, and the quality of downstream T- and B-cell priming that support IBV-specific immunity. Thus, the relevant logic chain for IBV is not simply that gut ecology “affects” respiratory vaccination, but that microbiome composition influences metabolite output; metabolite output helps condition the gut–lung axis; the gut–lung axis shapes respiratory immune priming; and this, in turn, contributes to variation in IBV vaccine uptake and durability [25,26,27,28].

A key translational point for IBV programs is that microbiome effects should be interpreted as response modifiers and not as replacements for established vaccine principles, such as strain matching, correct administration, schedule, and biosecurity. The microbiome perspective helps explain why birds managed under apparently similar protocols can exhibit heterogeneous responses. Early life gut ecology may shape baseline immune readiness, whereas later disruptions, such as diet shifts, anticoccidial programs, and stress, can alter metabolite profiles that influence germinal center quality and antibody maturation. Importantly, microbiome effects on antibody responses have been well documented across vaccine biology, supporting that gut microbial status can influence systemic responses to vaccination and providing credible mechanistic pathways for poultry translation [29]. However, much of this broader vaccine-biology evidence comes from non-IBV systems, and poultry-specific extrapolation should be interpreted cautiously.

Finally, IBV vaccination rarely occurs in isolation under field conditions; it is embedded in a landscape of respiratory pressure where pathogens such as Newcastle disease virus (NDV) and others may co-occur, and where gut ecology can be reshaped by infection itself. Evidence shows that NDV infection can alter the chicken gut microbiota with patterns dependent on NDV virulence, including loss of beneficial bacteria and expansion of potentially pathogenic taxa [30]. This interaction has implications for IBV vaccination because coinfection or recent NDV exposure can alter the baseline microbiome at the time of IBV vaccination or booster administration, potentially shifting immune tone and contributing to within-flock variation. Therefore, microbiome-guided strategies must explicitly account for respiratory disease ecology rather than treating vaccine response as a static trait.

## 4. Mechanisms Most Relevant to IBV Vaccine Uptake: Innate Priming, Germinal Centers, and Metabolite Context

For IBV vaccination, the microbiome-linked mechanisms most relevant to vaccine effectiveness are those that influence early innate priming and germinal center quality, because these steps determine the magnitude and durability of the humoral response that supports protection. The microbiome contributes to immune readiness through microbial structural cues and baseline stimulation that calibrates antigen presentation and cytokine tone. When these cues are absent or disrupted, vaccine priming may become weaker or misdirected. Evidence from broader vaccine–microbiome studies indicate that microbial status can influences responses to both mucosal and parenteral vaccines, supporting the plausibility that poultry respiratory vaccination may similarly be influenced by gut ecology [31]. This inference is based mainly on broader cross-species vaccine literature rather than direct IBV challenge–vaccination datasets. In the context of IBV, the practical goal is not to maximize inflammation, but to maintain an immune state that is sufficiently primed for effective antigen presentation while avoiding chronic inflammatory suppression or immune diversion.

Microbial metabolites, particularly SCFAs, provide a direct pathway through which gut ecology can reprogram immune-cell metabolism and differentiation relevant to antibody production. SCFAs can support B-cell biology and antibody production through metabolic and epigenetic mechanisms, and the broader literature highlights SCFAs as major mediators linking microbiota composition to humoral outcomes [32]. At present, this evidence is strongest in mammalian and general immunology literature, whereas direct poultry IBV validation remains limited. From a translational perspective, this suggests that variation in IBV vaccine responses may reflect differences in fermentative capacity and metabolite balance rather than taxonomy alone, which is important because metabolite-oriented readouts may be more stable and actionable than shifts in individual bacterial taxa.

However, metabolite effects are dose- and context-dependent, highlighting the need to avoid overly simplified supplementation strategies. Studies investigating the gut–lung axis in chickens demonstrate that microbiota modulation can enhance innate immunity, but it also underscores the need to align interventions with developmental timing and immune context rather than assuming uniform benefits from single compounds [33]. These avian data support biological plausibility, but they should not yet be interpreted as defining an established dosing framework for IBV-directed SCFA or metabolite interventions. In IBV programs, this suggests that strategies should prioritize stabilizing gut function and the metabolite environment during key vaccination windows, such as hatchery and early life priming stages, and booster periods, rather than relying on high-dose interventions that might unpredictably suppress key helper pathways required for germinal center maintenance.

Finally, findings from the IBV-microbiome association study provide a practical indication that immune cellular context (e.g., γδ T-cell-linked signals) may play a role in how gut ecology influences vaccine responsiveness [34]. These findings support a mechanistic model in which gut microbial composition shapes circulating metabolite cues, these cues influence systemic and respiratory immune programming through the gut–lung axis, and the resulting effects on antigen presentation, helper responses, and humoral priming condition IBV vaccine outcomes.

## 5. Practical Levers to Improve IBV Vaccine Response Consistency: Management Timing, Nutrition, and Microbiome-Support Tools

Because IBV vaccination is implemented at scale, the most useful microbiome-guided strategies are those that are simple, standardized, and compatible with hatchery and farm logistics. A key lever is timing alignment: minimizing major gut disruptions near important vaccination windows (e.g., immediately before or after priming or booster doses) when feasible, because microbiome disruptions can alter immune readiness and systemic inflammatory tone. This is not an argument to avoid necessary therapies; rather, it provides a framework for reducing avoidable stressors, such as abrupt feed transitions, unnecessary antimicrobial exposure, or severe enteric instability, that may contribute to response heterogeneity. The perspective is strengthened by the fact that NDV infection itself can alter gut microbiota, potentially shifting the immunometabolic baseline at the time of IBV vaccination. Such interactions highlight why respiratory disease ecology should be considered when interpreting vaccine response variability [35].

Nutrition is the most scalable microbiome lever, as it simultaneously shapes microbial fermentation and provides substrates for immune metabolism. In the context of IBV vaccination, the goal is to support a metabolite environment that enables effective priming and antibody maturation while minimizing excess inflammatory noise [36,37]. Adequate diet formulations, including amino acids that support barrier function and immune biosynthesis, trace minerals that support antioxidant defenses, and fermentable substrates that influence SCFA output, may influence the consistency of humoral responses in high-pressure environments. The gut–lung axis synthesis in chickens explicitly discusses how feeding strategies and microbiome modulation could strengthen mucosal immunity, which provides a clear conceptual basis for poultry-specific translation [38].

Microbiome-support tools, such as prebiotics, phytogenics, organic acids, and postbiotic-type preparations, should be presented as context-dependent adjuncts rather than universal solutions. The strongest scientific framing focuses on functional outcomes: barrier stabilization, reducing dysbiosis-associated inflammatory tone, and maintaining fermentative balance that produces beneficial metabolite profiles. Because the IBV vaccine–microbiome association has been demonstrated, intervention studies can be designed to test whether shifting gut ecology away from risk-associated signatures (e.g., higher *Escherichia–Shigella* OTUs) improves response uniformity under controlled conditions [22,39,40].

Finally, any practical lever should be evaluated against endpoints that are relevant for IBV vaccination: uniformity of antibody responses within the flock, durability across time, reduction in breakthrough respiratory signs during challenge periods, and reduction in secondary antimicrobial demand triggered by respiratory disease instability (Table 1). Importantly, the broader vaccine–microbiome literature provides evidence that microbiota can influence vaccine responses, making poultry-specific translation credible [41,42,43].

## 6. Biomarkers Tailored for IBV Programs: Making Microbiome-Guided Vaccination Measurable in Practice

**Figure 2:**
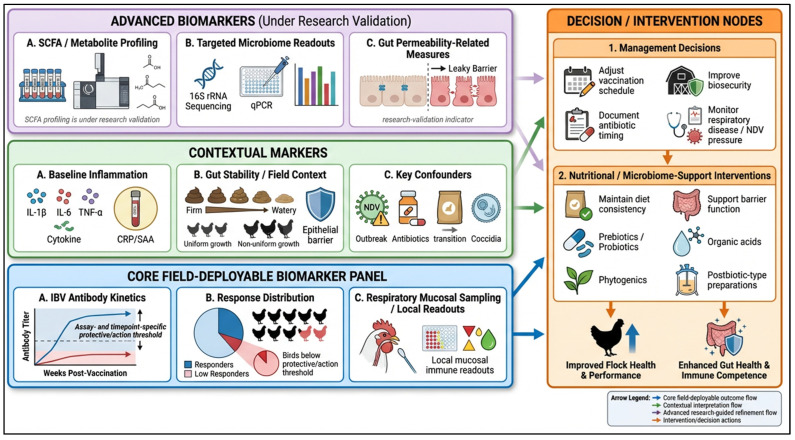
Tiered biomarker framework for predicting and monitoring IBV vaccine response under field conditions. Schematic of a tiered framework integrating core field-deployable indicators, contextual markers, and advanced biomarkers under research validation to guide decision-making and intervention planning for IBV vaccination programs in poultry. Created using https://www.figurelabs.ai, accessed on 3 March 2026.

In the context of IBV vaccination, the most useful biomarker strategy is a tiered monitoring panel that separates core field-deployable indicators from advanced research-validation layers (Figure 2). At its core are familiar vaccine outcome measures: antibody titers at standardized timepoints and, where feasible, functional correlates, such as neutralization or surrogate assays [44]. However, titers alone can be misleading when birds differ in baseline inflammation or when a subset of birds underperforms. For respiratory IBV programs, this core framework can be strengthened by incorporating respiratory mucosal sampling where feasible (e.g., tracheal or upper-airway samples) together with local immune readouts relevant to mucosal protection, rather than relying only on systemic antibody measures. Therefore, programs should evaluate not only mean titers but also response dispersion, such as the proportion of birds below a locally defined protective or action threshold based on the assay platform, vaccine program, and sampling timepoint, because field failures are often driven by pockets of low responders rather than uniformly weak responses [45,46,47,48,49,50,51,52,53].

To link microbiome function with IBV outcomes without requiring routine sequencing, metabolite proxies that indicate fermentative capacity can be considered as an advanced layer under research validation. For instance, SCFA profiles may be assessed in research contexts, alongside simple health indicators that reflect gut stability, such as fecal consistency trends and growth uniformity during critical periods [54,55]. Where included, gut permeability-related measures or other mechanistic metabolite readouts should likewise be interpreted as research-validation indicators rather than routine field standards at present. This approach is mechanistically justified because SCFAs and other metabolites are repeatedly highlighted as key mediators through which microbiota influence immune responses. Additionally, studies examining the gut–lung axis suggest that microbiome modulation can shape systemic and respiratory immune readiness in chickens [56,57,58,59]. For higher-resolution investigations, such as breeder flocks, high-value operations, and research trials, targeted microbiome assays can be included to test whether IBV-associated signatures reported in controlled studies are replicated under local field conditions [44,60,61].

Because NDV and other respiratory pressures can reshape gut ecology, the biomarker framework should explicitly capture key confounders, including recent NDV exposure or outbreak indicators, recent antimicrobial pulses, major feed transitions, and coccidial cycling intensity [62,63]. In addition, longitudinal biomarker studies should distinguish pre-vaccination baseline microbiome features from post-vaccination shifts, because immune activation during or after vaccination may itself alter gut microbial composition and metabolite patterns. NDV infection has been shown to alter gut microbiota composition in a virulence-dependent manner, implying that microbiome status at the time of vaccination may reflect recent infection history. Interpreting IBV response variation without accounting for these covariates may lead to inaccurate conclusions [64,65]. In practice, a concise confounder checklist can often add more interpretive value than high-dimensional sequencing alone.

Finally, reporting should be standardized to enable comparisons across trials and farms. At a minimum, studies should document the vaccine strain and schedule, bird age, sampling time points, antimicrobial exposures, changes in diet composition, and respiratory disease events, along with the biomarker panels used. Without harmonized reporting and clearer separation between actionable core indicators and exploratory or research-validation biomarkers, these strategies are likely to remain descriptive and difficult to translate into consistent adjustments to vaccine programs.

## 7. Translational Trial Design Centered on IBV: From Association to Actionable Interventions

To translate IBV microbiome associations into actionable recommendations, trials must be designed to distinguish true intervention effects from confounding factors such as management practices and disease pressure. The IBV study demonstrating an association between cecal microbiota composition and vaccine response provides a strong starting point; however, translation requires interventions that intentionally shift microbiome function and assess whether IBV vaccine response distribution improves [66,67]. A practical and high-value approach is a stratified field trial in which flocks are grouped by baseline “risk context” (e.g., recent antimicrobial exposure, enteric instability, or recent NDV activity. Each group would then receive a defined, standardized microbiome-support intervention during a specified vaccination window, with microbiome sampling performed at baseline before vaccination and at defined post-vaccination timepoints, followed by consistent follow-up for immune and health endpoints [68,69,70].

Endpoint selection should be IBV-specific and relevant to field conditions, such as antibody kinetics at defined post-vaccination timepoints, the proportion of low responders, trends in respiratory clinical scores during challenge periods, and secondary antimicrobial use triggered by respiratory outbreaks [71,72]. Where feasible, trials should include a controlled challenge component or at least standardized exposure-risk periods, as real-world disease pressure is uneven and can significantly influence outcomes. Trials must also document and adjust for NDV dynamics, as NDV infection can alter gut microbiota and thus modify both the exposure (microbiome status) and the outcome (respiratory disease and immune tone) [73,74]. Without accounting for this, trials may wrongly attribute response differences to interventions that merely coincided with changes in respiratory pathogen pressure.

Mechanistic sampling should be tiered to match feasibility. A core panel, such as titers, low-responder fraction, and basic health or performance, should be collected. Additionally, optional mechanistic layers, such as metabolite profiling and targeted microbiome assays, can be applied to a subset to confirm whether the intervention influenced the system through the hypothesized pathways. This approach prevents overburdening field trials while still generating the mechanistic evidence needed for credible recommendations. Evidence from gut–lung axis studies suggest that microbiome modulation can influence mucosal immunity in chickens, which provides a clear mechanistic rationale for designing sampling based on immune readiness and respiratory outcomes [75,76,77].

Finally, minimum reporting standards should be clearly defined so that studies can be compared and synthesized (Table 2). Reports should include vaccine strain(s) and schedules, age, housing conditions, diet composition and transitions, antimicrobial and anticoccidial exposures with timing, respiratory outbreak logs (including NDV indicators), and all sampling timepoints. This level of reporting is essential because microbiome–vaccine effects are highly context dependent; rigorous reporting can provide compelling and generalizable findings that can guide vaccination programs across diverse production systems.

## 8. Limitations, Confounders, and Ethics

**Figure 3:**
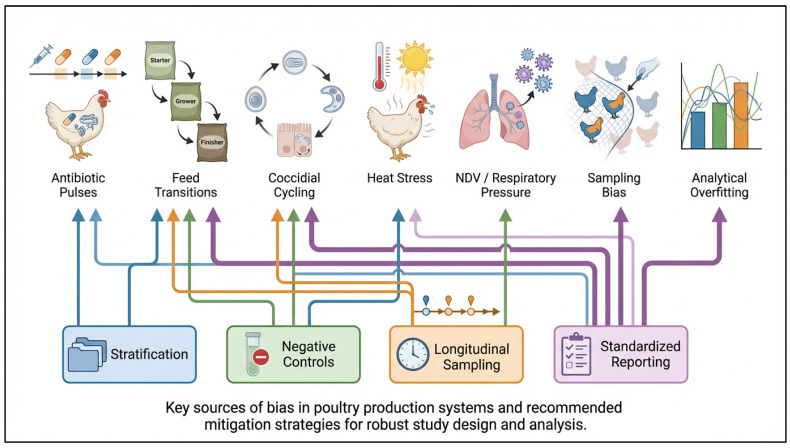
Confounders and failure modes that distort microbiome–IBV vaccine associations. Conceptual overview of key biological, environmental, and analytical factors that can bias or obscure associations between the microbiome and IBV vaccine responses. Corresponding mitigation strategies (e.g., stratification, controls, longitudinal sampling, and standardized reporting) are highlighted to improve study robustness. Created using https://www.figurelabs.ai, accessed on 3 March 2026.

Microbiome-guided vaccination for IBV is highly vulnerable to confounding because the same field factors that reshape gut ecology can also independently influence vaccine outcomes. The most significant confounders include antibiotic pulses, abrupt dietary transitions, coccidial cycling, heat stress, and concurrent respiratory pressure. Each of these can simultaneously alter microbiome function, baseline inflammation, and the likelihood of IBV challenge exposure (Figure 3) [78,79]. NDV introduces a particularly important poultry-specific confounder, as infection has been shown to shift gut microbiota in a virulence-dependent manner. Consequently, baseline microbiome state at the time of IBV vaccination may reflect recent or ongoing respiratory disease dynamics rather than a stable trait of the flock. If such confounders are not recorded and adjusted for, microbiome signatures may be misinterpreted as causal drivers of vaccine response when they are actually indicators of upstream stress or pathogen pressure [80].

A second limitation is the causal gap between association and intervention, including uncertainty about temporal directionality, because some microbiome changes observed after vaccination may reflect consequences of immune activation rather than true baseline predictors of vaccine performance. The study by Borey et al. [23] linking cecal microbiota composition to IBV vaccine response provides an important foundation. However, association alone cannot justify specific additive recommendations unless an intervention demonstrably shifts microbiome or metabolite features and improves the distribution of IBV responses, particularly by reducing the low-responder tail. This highlights the importance of responder-stratified designs. By identifying at-risk subgroups, such as flocks with recent antibiotic exposure, enteric instability, or NDV activity, and testing targeted stabilization strategies, trials can move from correlation toward actionable causation without relying on unrealistic routine sequencing [81]. Overinterpretation is also a concern when microbiome effects are considered universally beneficial. In addition, the present discussion is centered mainly on bacterial community structure and metabolite function; however, non-bacterial components of the microbiome, including the virome, mycobiome, and bacteriophages, may also influence immune tone and vaccine-response variability, although their roles in poultry IBV systems remain insufficiently resolved. Metabolite biology, including SCFAs, is context-dependent; therefore, single-compound supplementation may be beneficial in one immunological context but neutral or counterproductive in another, particularly if it alters helper pathways required for germinal center quality [82].

Minimizing nonessential disruption around IBV vaccination windows does not imply delaying clinically necessary therapy. Rather, it provides a decision framework that improves planning and recordkeeping, so vaccination is not repeatedly scheduled during periods of significant gut instability when avoidable. Similarly, supplement strategies should be framed as status-aware and dose-disciplined, with emphasis on standardized composition and transparent reporting to prevent the field from becoming saturated with non-comparable claims. Data practices are also important; because outbreak logs and treatment records are required to interpret microbiome–vaccine relationships, programs should ensure confidentiality and focus on improvement rather than blame. This approach can increase participation and improve the quality of evidence generated for flock-level decision making.

## 9. Conclusions

IBV respiratory vaccination provides a useful model for microbiome-guided vaccine optimization in food animals because it lies at the intersection of respiratory immunity, gut microbial ecology, and the complexity of field-level management. Current evidence indicates that cecal microbiota composition is associated with variation in IBV vaccine responsiveness, while the chicken gut–lung axis offers a plausible mechanistic framework through which gut ecology and microbial metabolites may influence respiratory immune readiness. Taken together, the reviewed literature suggests that vaccination effectiveness is shaped not only by vaccine-related factors, but also by peri-vaccination stressors, antibiotic exposure, diet transitions, and concurrent respiratory disease pressures, particularly NDV, which can modify gut microbiota and confound immune outcomes. In practical terms, the most immediate translational opportunities lie in stabilizing the peri-vaccination window, maintaining nutritional consistency and gut barrier support, and using microbiome-targeted tools such as prebiotics, organic acids, phytogenics, and postbiotic-type preparations as context-dependent adjuncts rather than universal solutions. Overall, the priority for the field is not routine sequencing, but the development of implementable programs that improve response uniformity, reduce low-responder subpopulations, and strengthen flock resilience under commercial conditions. Further progress will depend on well-designed intervention trials, flock-level monitoring frameworks, and minimum reporting standards that enable consistent evaluation of microbiome- and metabolite-linked strategies and their translation into actionable IBV vaccination programs. While this review primarily focuses on poultry and IBV vaccination, the underlying concepts linking microbiome composition, host immune responsiveness, and peri-vaccination management are likely relevant across food animal systems. In ruminants, emerging insights into the rumen–immune interface and microbiome-mediated modulation of vaccine responses suggest that similar principles may apply, although species-specific physiology and production conditions will influence their practical implementation. Dedicated research in ruminant systems remains limited and represents an important direction for future investigation.

## Figures and Tables

**Table 1:** Practical levers for improving IBV vaccine response consistency through microbiome and immunometabolism.

Lever (Field-Deployable)	Proposed Mechanism (Microbiome/Metabolites + Host)	Target Immune Step Most Relevant to IBV	Typical Outcomes to Measure	Evidence Strength (for IBV Framing)	Field Constraints/Notes
Peri-vaccination “stability window” management (avoid major stressors 3–7 d around priming/boosting)	Reduces dysbiosis and inflammatory noise that can impair antigen presentation and germinal center quality; stabilizes gut–lung axis immune readiness	Innate priming; T-helper support; germinal center initiation	Antibody kinetics; low-responder fraction; respiratory signs during challenge windows; antimicrobial courses	Moderate (conceptual + multi-species evidence); strengthened by chicken gut–lung axis literature	Requires coordination with feed changes, thinning, transport, vaccination day logistics
Antibiotic timing discipline (avoid nonessential antibiotics around vaccination window; document unavoidable exposures)	Reduces peri-vaccination antibiotic-driven dysbiosis that may blunt innate priming, alter microbial metabolite availability, and impair antigen presentation, B-cell support, and antibody maturation; unavoidable exposures should be recorded to interpret response heterogeneity	Priming and memory formation; antibody maturation	Antibody titers/avidity; durability; relapse/breakthrough clusters	High (causal evidence in mammalian vaccine models); translational plausibility for poultry	Welfare-first: clinically required treatment must be applied; emphasize timing/recording rather than prohibition
Diet transition smoothing (stepwise energy/protein changes instead of abrupt switches)	Limits abrupt shifts in substrate supply that destabilize fermentation, barrier integrity, and inflammatory tone, thereby helping maintain a peri-vaccination metabolite environment compatible with effective immune priming	Immune readiness; reduced baseline inflammation	Uniformity of response; gut stability proxies; growth uniformity; respiratory disease treatment rates	Moderate (mechanistic plausibility + poultry immunometabolism synthesis)	Depends on feed mill and farm planning; may be easier in integrated systems
Fermentable fiber/prebiotic strategy (e.g., controlled inclusion of fermentable substrates)	Promotes stable fermentation and metabolite outputs (SCFAs), potentially supporting B-cell metabolism and mucosal immune tone	Germinal center efficiency; antibody magnitude/durability	Antibody kinetics; low-responder fraction; SCFA profiles (research); FCR/ADG	Moderate (mechanistic evidence linking SCFAs to humoral immunity; context dependent)	Must avoid wet litter/enteritis risk from excessive fermentables; response can vary based on baseline microbiome
Targeted amino-acid adequacy (threonine/methionine/cysteine emphasis around key windows)	Supports mucin/barrier and immune biosynthesis; improves redox and methylation capacity; indirectly stabilizes microbiome	Barrier-immune coupling; sustained antibody production	Antibody kinetics; gut health indicators; growth uniformity	Low–Moderate (strong biological plausibility; IBV-specific data limited)	Benefit highest when correcting marginal adequacy; avoid making single-nutrient claims without trials
Trace mineral optimization (bioavailable forms) (Zn/Se/Cu status-aware)	Supports antioxidant defense and innate signaling; reduces oxidative stress that can distort immune responses	Priming and response quality	Antibody response distribution; inflammatory markers; performance	Low–Moderate (plausible; requires status-aware framing)	Risk of excess; baseline deficiency status determines benefit; ensure compliance with local regulations
Organic acids/acidifiers	May stabilize luminal pH and microbial ecology, reduce opportunist expansion, and support barrier function, thereby lowering dysbiosis-associated inflammatory diversion during vaccine priming	Immune readiness; reduced dysbiosis-associated immune diversion	Response uniformity; diarrhea/enteritis incidence; performance	Low–Moderate (variable across systems; supportive rationale)	Dose and formulation matter; may interact with water line management and palatability
Phytogenics (standardized extracts)	May modulate inflammatory tone, oxidative stress, and microbial community balance, thereby indirectly supporting antigen presentation quality and reducing non-specific immune diversion	Priming quality; reduced immune diversion	Antibody kinetics; clinical respiratory signs; performance	Low (heterogeneous formulations; IBV-specific evidence sparse)	Product variability is a major issue; require specification of active components and dose
Postbiotic-type preparations (inanimate microbial components/metabolites)	Provide standardized non-viable microbial components and metabolites that may support barrier integrity and immune modulation without the viability variability associated with live probiotics	Peri-vaccination immune stability	Low-responder fraction; gut health; respiratory treatment rates	Low–Moderate (strong conceptual fit; needs IBV-anchored trials)	Must report composition/inactivation method; avoid vague probiotic-like labeling without characterization
NDV/respiratory pressure control as a microbiome confounder (biosecurity + outbreak logging)	NDV infection can shift gut microbiota, altering immunometabolic baseline and confounding IBV response interpretation	Confounder control rather than direct immune step	Reduced variance in response; cleaner attribution of interventions	High (NDV→microbiome alteration evidence)	Not a supplement; requires surveillance, outbreak logs, and vaccination program coordination
Responder-stratified interventions (apply gut-support only for at-risk groups)	Targets resources for birds likely to respond poorly (recent antibiotics/enteritis/NDV activity), increasing cost-effectiveness and signal	Improves population distribution of response	Proportion below threshold; durability; antibiotic-use metrics	Moderate (strong methodological value; aligns with association evidence in IBV response)	Requires simple risk scoring and recordkeeping; avoids “treat everyone” inefficiency

**Table 2:** Minimum reporting checklist for IBV microbiome–vaccine trials in poultry.

Domain	Minimum Items to Report (Essential)	Significance for IBV Microbiome Framing
Birds and management	Line/breed; sex (if relevant); flock type (broiler/layer/breeder); stocking density; house type; temperature/heat stress periods	Genetics and stress shape baseline immunity and gut ecology; must be accounted for when interpreting response distributions
Vaccine details	IBV vaccine type (live/inactivated); strain(s); dose; route (spray/ocular/drinking water/injection); schedule and exact ages; co-administered vaccines (NDV, etc.)	Vaccine strain/route drives mucosal priming; co-vaccination and schedule timing can interact with immune tone and confound interpretation
Administration quality	Waterline sanitation; vaccine uptake monitoring (dye checks or consumption proxy); spray quality/particle size (if used); handling time	Variation in delivery can mimic microbiome effects by creating low-responder pockets unrelated to biology
Diet and transitions	Full diet formulation (at least key specs); feed changes (date and composition shift); coccidiostat program; mycotoxin binders (if used)	Diet is the strongest microbiome driver; transitions are major confounders around vaccination windows
Antimicrobial exposures	Drug class; indication; dose; duration; timing relative to vaccination (days pre/post); metaphylaxis vs therapy	Antibiotic-driven dysbiosis can alter vaccine responses; timing is a primary effect modifier
Respiratory disease ecology	NDV activity indicators/outbreak logs; other respiratory agents if monitored; mortality spikes; clinical scoring	NDV can shift gut microbiota and confound IBV response interpretation; must be logged explicitly
Sampling strategy	Sample type (cecal content/feces; tracheal swab; blood); timepoints (baseline + defined post-vaccination days); number per flock; pooling rules	Enables interpretation of cause versus consequence and supports consistent comparison across trials
Microbiome methods (if used)	Extraction kit; sequencing target (16S/shotgun); platform; batch randomization; negative controls; bioinformatics pipeline and thresholds	Without controls and pipeline transparency, microbiome associations are not reproducible
Metabolite methods (if used)	SCFA measurement method; sample handling/storage; normalization approach	Metabolites may mediate effects more directly than taxa; poor handling invalidates interpretation
Immune endpoints	Antibody titers at fixed timepoints; proportion below threshold; (optional) functional assays; (optional) cellular markers (e.g., γδ T-cell related measures)	IBV “breaks” often driven by low-responder pockets; association studies suggest cellular context may be important
Clinical/protection endpoints	Respiratory signs scoring; lesion scoring (if available); production outcomes (FCR/ADG/egg metrics); culls; mortality	Keeps the study anchored to real protection/performance rather than biomarkers only
Antibiotic-sparing endpoints	Number of antibiotic courses; days on therapy; proportion treated; retreatment rate	Essential for demonstrating practical value beyond immunology
Statistics	Provide standardized non-viable microbial components and metabolites that may support barrier integrity and immune modulation without the viability variability associated with live probiotics	Improves robustness and reduces false-positive interpretation.

## Data Availability

No new data were created or analyzed in this study.
